# *Ainsliaea daheishanensis* (Asteraceae): a new species from China

**DOI:** 10.3897/phytokeys.138.38566

**Published:** 2020-01-10

**Authors:** Yulan Peng, Chenxuan Yang, Yan Luo

**Affiliations:** 1 Key Laboratory of Mountain Ecological Restoration and Bioresource Utilization & Ecological Restoration Biodiversity Conservation, Chengdu Institute of Biology, Chinese Academy of Sciences, Chengdu, Sichuan 610041, China; 2 Key Laboratory of Sichuan Province, Chengdu Institute of Biology, Chinese Academy of Sciences, P.O. Box 416, Chengdu, Sichuan 610041, China; 3 Gardening and Horticulture Department, Xishuangbanna Tropical Botanical Garden, Chinese Academy of Sciences, Menglun, Mengla, Yunnan 666303, China

**Keywords:** Asteraceae, *Ainsliaea daheishanensis*, China, new species

## Abstract

In this work, we describe a new species, *Ainsliaea daheishanensis* Y.L.Peng, C.X.Yang & Y.Luo, based on morphological traits. The new species was discovered in the mountains of Yunnan, near the border between Myanmar and China. The new species differs from the phenotypically closely-related *Ainsliaea foliosa* Handel-Mazzetti in the morphology of the leaf veins and phyllaries, those having a protruding abaxial reticulate pattern in the lower and median part of stem with white hairs and narrow inner phyllaries. A key to the three closed *Ainsliaea* species occurring in China is provided.

## Introduction

*Ainsliaea* DC., first described by de Candolle (1838), belongs to the subfamily Mutisioideae, tribe Mutisieae. The genus *Ainsliaea* is a monophyletic group as supported by molecular data (Mitsu et al. 2008) and it includes 50 estimated species distributed in Afghanistan, Bangladesh, Bhutan, China, India, Indonesia, Japan, Korea, Myanmar, Nepal, Pakistan, Philippines, Thailand and Vietnam (Freire 2007, Gao et al. 2011). In addition, in recent years, new species have been reported in China and Vietnam (Qian 2000, Freire 2002, Wang et al. 2010). China is the centre of diversity for *Ainsliaea*. Forty of them are distributed in China, including 28 endemic species (Wang et al. 2010, Gao et al. 2011). However, some species are restricted only to a very narrow area of Sichuan and Yunnan.

During our fieldwork on the border between China and Myanmar, we found a novel and undescribed species of *Ainsliaea* in the Dahei Mountain in Menglian County. This new species is easily distinguishable from other taxa in the *Ainsliaea* genus by the protruding white hairy reticulate veins on the lower surface of leaves and narrow inner phyllaries. Here, we name it as *A. daheishanensis* Y.L.Peng, C.X.Yang & Y.Luo, sp. nov. and we describe its morphology, based on the living plants in the field and several collections in the herbarium.

## New species description

### Ainsliaea daheishanensis

Taxon classificationPlantaeAsteralesAsteraceae

Y.L.Peng, C.X.Yang & Y.Luo sp. nov.

93B0533E-F567-5CD6-9B1E-356D8E125E70

urn:lsid:ipni.org:names:77204220-1

Figs 1 , 2 , 3

#### Diagnosis.

This new species is similar to *Ainsliaea foliosa* Handel-Mazzetti and *A. latifolia* (D. Don) Schultz Bipontinus, but it differs from them in its solitary white hairy reticulate veins on the abaxial surface of the lower part of the leaves and on the narrow inner phyllaries.

#### Type.

**Figure 1.**
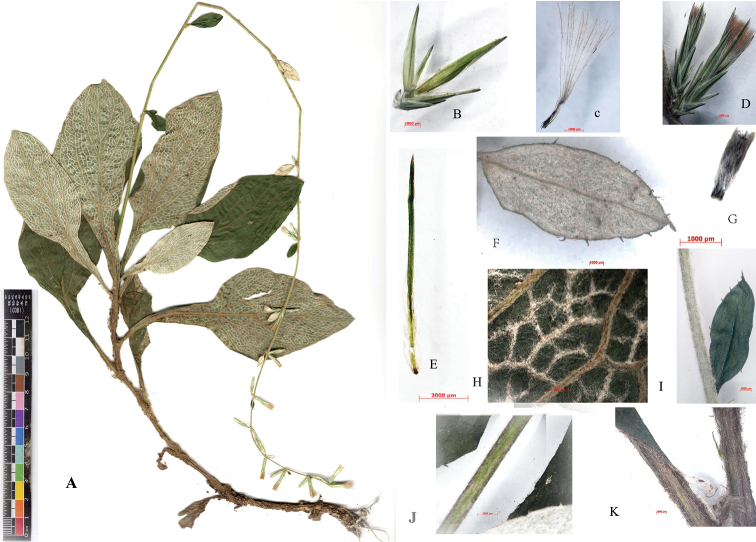
*Ainsliaea daheishanensis* Y.L. Peng, C.X. Yang & Y. Luo, sp. nov. **A** habit **B** involucres **C** pappus **D** inflorescences **E** inner phyllary **F** upper leaf **G** magnified achene **H** magnified abaxial surface of median part leaves of the stem **I–J** upper part of stem and leaves, respectively **K** lower part of the stem. Photo taken by Y.L. Peng based on the holotype.

China: Under oak forest, Yunnan Province: Menglian County, Lafu village, Mountain Dahei, 22.102733°N, 99.40731°E, elevation 2092–2300 m, 14 January 2019, Y.L. Peng & C.X. Yang SE02248 (holotype CDBI!, isotype HITBC!) (Figure 1).

#### Description.

Plants perennial, herbaceous, 60–80 cm tall. Stems erect, unbranched, villous. Leaves alternated in lower to median part of the stem. Petiole 4–6 cm long, large winged, gradually reducing, villous, leaf blades papery, palmate-pinnate veined, ovate to elliptic, 8–10 × 2–4 cm, apex acute, base abruptly constricted into winged petiole, margin obscurely callose-denticulate, slightly discoloured, upper surface green, sparsely strigose, subglabrous palmate-pinnate veined, lower surface pale with evident reticular veins densely covered by thick white hairs, the remaining part of the lower surface light green and subglabrous. Upper leaves ovate to elliptic, 1–3 × 0.5–1.5 cm, upper surface green-olivaceous, subglabrous palmate-pinnate veined, lower surface densely covered in thick white hairs. Capitula sessile and distantly spaced upwards to the inflorescence axis; disposed in spikes, involucre 6-seriate, cylindrical, ca. 15 × 5 mm; phyllaries papyraceous, glabrous, or sparsely pilose, outer phyllaries ovate, acute, ca. 2 × 1 mm; inner phyllaries linear-oblong to lanceolate, acute, mid-vein dark green, margin pale to pale green. ca. 15 × 0.3 mm. Florets ca. 3–4, flowers not present. Achenes ca. 2–3 mm, densely pilose, pappus reddish-brown, ca. 7 mm long.

**Figure 2.**
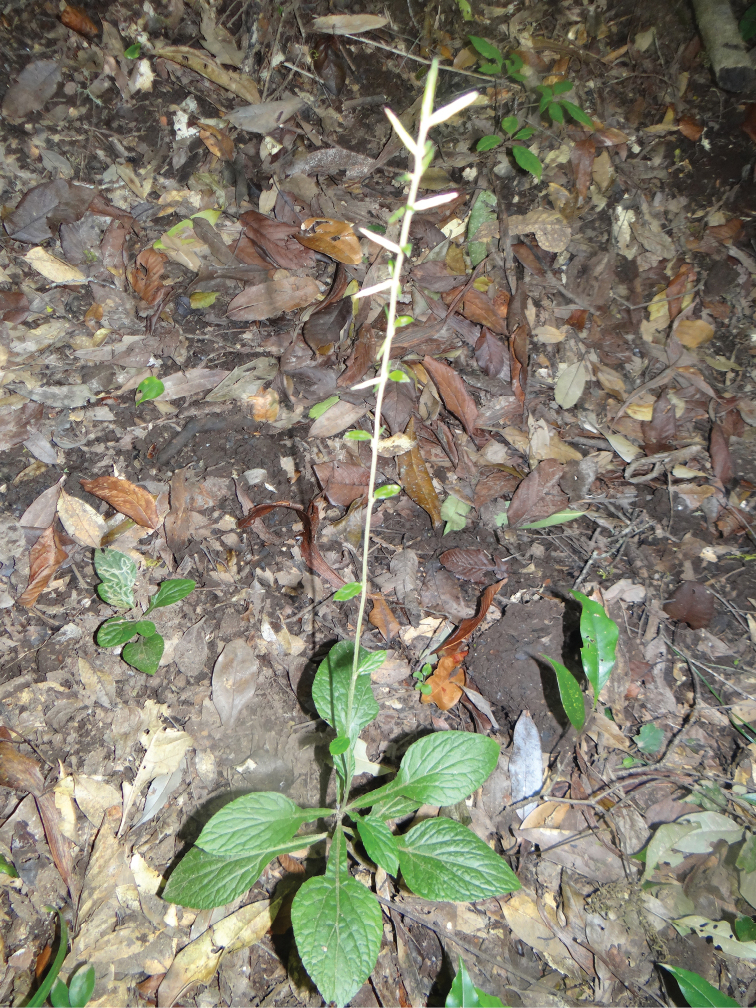
The whole plant *Ainsliaea daheishanensis* Y.L.Peng, C.X.Yang & Y.Luo, sp. nov. in its habitat (under evergreen forest).

**Figure 3.**
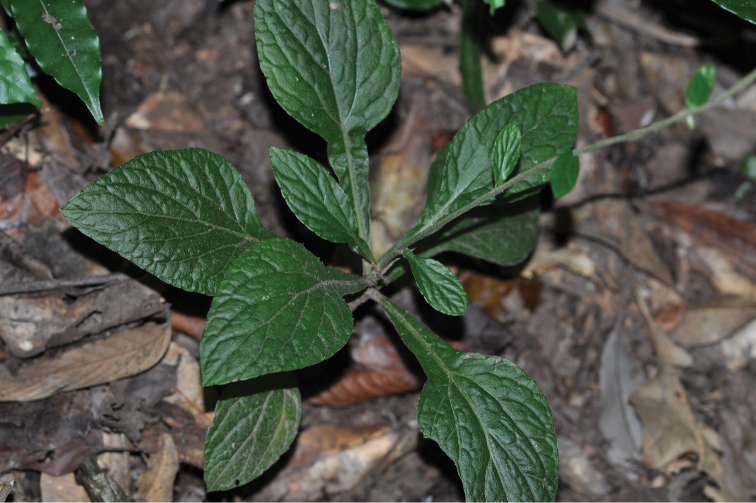
The lower part of the plant *Ainsliaea daheishanensis* Y.L.Peng, C.X.Yang & Y.Luo, sp. nov. in the field.

#### Etymology.

The new specific epithet “daheishanensis” refers to the name of the Dahei Mountain, located at the border between China and Myanmar, where the novel species was discovered.

#### Phenology.

Flowering was not observed, fruiting in November-March.

#### Distribution and habitat.

**Figure 4.**
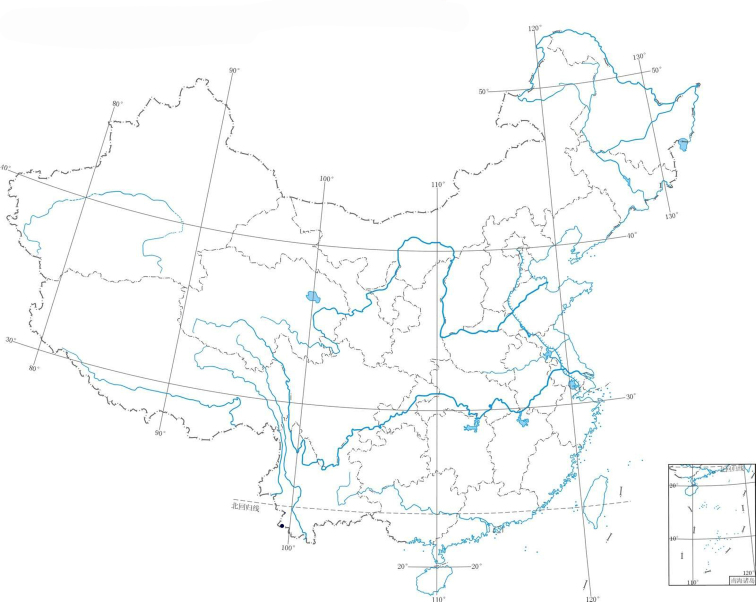
Distribution of *Ainsliaea daheishanensis* Y. L. Peng, C. Y. Yang & Y. Luo. • *A. daheishanensis*.

*Ainsliaea daheishanensis* is only known from the type collection cited above, at 2100–2300 metres altitude, under evergreen forests (Figure 4). The other examined specimens e.g. Y.Y.Qian 2818, have no detailed collection information; they are only found in Yunnan Province.

#### Additional material examined.

China. Yunnan: Menglian County, Lafu village, under evergreen forest, elevation 2300 m, 8 November 2010, S.S. Zhou 7755 (HITBC).Yunnan Province: Menglian County, 8 March 1993, Y.Y.Qian 2818(HITBC).

#### Discussion.

This new species is mostly similar to *Ainsliaea foliosa* in the broadly winged leaves that are loosely aggregated near the median part of the stem and having an ovate blade. *Ainsliaea daheishanensis* can be distinguished from *A. foliosa* by its protruding white reticulate veins on the abaxial surface of the lower part of cauline leaves, which is covered with thick white hairs and by the innermost phyllaries that are narrow and slightly shorter than the crown hairs (Table 1) (Fig. 1A, B, E, H). *Ainsliaea daheishanensis* resembles *A. latifolia* by its leaves and inflorescences. Both species have ovate to elliptic leaf blades, with long and winged petioles and the capitula are arranged in spikes. These similarities between the two species led some researchers to wrongly identify the specimens of *Ainsliaea daheishanensis* as *A. latifolia*. Morphologically, *Ainsliaea daheishanensis* differs from *A. latifolia* in the position of the leaves appearing above the base of the stem (vs. a basal rosette in *A. latifolia*), and in the evident reticulate veins of the abaxial surface of leaves with thick white hairs, mainly occurring in the reticulate veins. The abaxial surface of *A. latifolia* leaves is densely covered with white fluff, mixed with long stiff hairs of the same colour. A key to the three closely related *Ainsliaea* species in China is provided below.

**Table 1.** List of the morphological differences amongst *Ainsliaea daheishanensis*, *A. foliosa* and *A. latifolia*.

**Characters**	***Ainsliaea daheishanensis***	***Ainsliaea foliosa***	***Ainsliaea latifolia***
Leaf arrangement patterns	Alternated in lower and median part of stem.	Loosely aggregated or occasionally alternated in median part of stem.	Basally clustered, rosulate.
Leaf morphology	Lower surface with obvious reticular veins, which are covered with thick white hairs.	Lower surface with sparse trichomes and obscure reticular veins	Lower surface densely covered with white fluff, mixed with long, slightly stiff hair of the same colour.
Petioles	4–6 cm, obviously shorter than leaf blade.	2.5–5 cm, almost equal or shorter than leaf blade.	(2) 4–9(11) cm, almost equal in length to leaf blade.
Capitula	Sessile, 1–3 clustered, arranged in spikes, 3–4 flowered.	Subsessile or shortly pedunculate, arranged in racemes or spikes, 4– or 5–flowered.	Subsessile or shortly pedunculate, (1 or) 2–4 clustered, arranged in spikes or panicles, 3-flowered.
Involucre	6 to 7-seriate, cylindrical, 8–10× ca. 4 mm; phyllaries papyraceous; outer phyllaries ovate, acute, 2–3 × ca. 1 mm, inner phyllaries linear-oblong, acute, mid-vein usually green, 15× 0.3 mm, slightly longer than the pappus.	Involucre 4-seriate, subleathery, outer phyllaries broadly ovate, 2.5–3 mm, inner phyllaries ovate to elliptic, apex purple, mid-vein usually dark green, 10 × 0.8 mm, evidently shorter than the pappus.	5 to 7-seriate, cylindrical, 8–10 × ca. 4 mm; phyllaries papyraceous; outer phyllaries ovate, acute, 2–3× ca. 1 mm, apically strigose; inner phyllaries linear-oblong, acute, mid-vein usually dark, 7–12 × ca. 1 mm, shorter than the pappus.

##### Key to *Ainsliaea daheishanensis*, *A. foliosa* and *A. latifolia*

**Table:** 

1	Leaves loosely aggregated or occasionally alternated in the median part of the stem, abaxial surface subglabrous, sparsely or partially hairy	**2**
–	Leaves basally clustered, rosulate, the abaxial surface densely covered with white fluff	***Ainsliaea latifolia***
2	Abaxial surface of leaves with recognisable reticular veins, thick white hairs only on the reticular veins, involucre 6 to 7-seriate; inner phyllaries linear-oblong, acute, apex green, mid-vein usually green, slightly longer than the pappus	***Ainsliaea daheishanensis***
–	Reticular veins of the abaxial surface of the leaves obscure, scattered with trichomes, involucre phyllaries 4-seriate, subleathery, inner phyllaries ovate to narrowly elliptic, apex purple, 0.8–1.1 cm, mid-vein usually dark green, noticeably shorter than the pappus	***Ainsliaea foliosa***

## Supplementary Material

XML Treatment for Ainsliaea daheishanensis
